# Water Management for Sustainable Irrigation Systems Using Internet-of-Things†

**DOI:** 10.3390/s20051402

**Published:** 2020-03-04

**Authors:** André Glória, Carolina Dionísio, Gonçalo Simões, João Cardoso, Pedro Sebastião

**Affiliations:** 1 ISCTE—Instituto Universitário de Lisboa, 1649-026 Lisbon, Portugal; cadoo@iscte-iul.pt (C.D.); garss@iscte-iul.pt (G.S.); jmbco1@iscte-iul.pt (J.C.); pedro.sebastiao@iscte-iul.pt (P.S.); 2 Instituto de Telecomunições, 1049-001 Lisbon, Portugal

**Keywords:** Internet of Things, Wireless Sensor Networks, sustainability, LoRa, ESP32, water savings

## Abstract

This paper introduces a new way of managing water in irrigation systems, which can be applied to gardens or agricultural fields, replacing human intervention with Wireless Sensor Networks. A typical irrigation system wastes on average 30% of the water used, due to poor management and configuration. This sustainable irrigation system allows a better efficiency in the process of irrigation that can lead to savings for the end user, not only monetary but also in natural resources, such as water and energy, leading to a more sustainable environment. The system can retrieve real time data and use them to determinate the correct amount of water to be used in a garden. With this solution, it is possible to save up to 34% of water when using sensor data from temperature, humidity and soil moisture, or up to 26% when using only temperature inputs. Besides a detailed system architecture, this paper includes a real case scenario implementation and results discussion.

## 1. Introduction

Research and implementation in the Internet-of-Things (IoT) fields and in topics such as Smart Cities, Agriculture and Sustainability have been growing in the past years [1].

As S. Routray [2] said “Green technologies and energy efficient process in IoT are well researched areas in which optimization of resources takes the central position”. IoT is impacting the world, creating new markets and innovation, as it connects a wide range of networks in both economy and society, requiring innovation in information, communications and regulations [3]. This emerging concept aims to disruptively enhance the efficiency, sustainability, and safety of cities, with integrated infrastructure and services, that allows a monitoring and management using intelligent devices and systems [4,5].

A big part of cities nowadays is their focus on creating green spaces, not only for their recreational purpose, but also because it helps reduce the carbon footprint and overall temperatures of cities, but the maintenance of those spaces includes usage of huge amounts of water.

Automatic irrigation solutions, based on manual configured controllers, composed of timers and irrigate every day at the same time and duration, are the basic solution and the go-to choice when building a new garden. This type of solution is still the main choice in around 80% of the irrigation projects due to low prices and simplicity, but this type of solution has a poor efficiency when it comes to water management; it is common to see them start the irrigation process in periods of rain, excessive heat or strong winds, conditions that contribute to the waste of water. Further, this type of solution is unable to understand if problems with the system, such as pipe leaks or broken valves, occur, contributing to garden flooding, not only killing the garden but also making it inaccessible to users.

Sustainability is nowadays directly linked to innovation, since all around the world, people are becoming more concerned with climate changes, water shortage, clean energies and other challenges that require the development of new products, business and services that can lead to advances not only in environmental dimensions but also in social and economic dimensions [3]. Only with innovation we are able to fight the ongoing process where natural resources, such as water, are starting to vanish and material goods prices, like energy, are at an all-time highs. Combining IoT, sustainability and Machine Learning algorithms is possible to create more efficient processes and achieve a disruptive innovation that in the medium to long term will allow consumption reduction in natural and material resources.

At this moment, water shortage is something that is rising every day, affecting more and more people. Approximately 70% of the fresh water used in many parts of the world is going for irrigation related activities, from agricultural fields to gardens, and according to [6], 30% of this water is being potentially wasted due to environmental conditions or from the cycles not being optimized for the types of plants. This waste is not only influencing the costs for the consumer but also is reducing the health of the fields, in either under or over irrigation.

To achieve the goal of reducing the waste of water in irrigation systems, disruptive solutions need to be the go-to solutions. Instead of another smart irrigation controller, our solution is a Sustainable Irrigation Solution, that not only controls the irrigation system, but also analyzes all the system components, from the pipes that supply the water to the sprinklers, in order to analyze potential leaks or malfunctions, improving efficiency and reducing costs in a sustainable and circular economy fashion.

Using Wireless Sensor Networks (WSN), directly in the garden to collect real time data to check the current conditions of the garden and crossing these information with weather forecast, evapotranspiration and garden specifications, our proposed solution is able, through artificial intelligence algorithms, to predict/calculate the real water needs for that particular garden and adjust the irrigation controller without human interaction. It is also able to detect how weather conditions, such as rain or strong winds, may affect the irrigation process, scheduling the best time to start. We stand out from other solutions is that we do this analysis per irrigation zone and not in the garden as a whole, since gardens can have different characteristics in their constitution, having also different hydric needs.

Combining this real time analysis with the use of the correct irrigation times, the detection of system malfunctions in the pipes and other materials, and the ability to control the entire system remotely through a mobile dashboard, we are able to reduce the waste of water in up to 30%, but also reduce the costs of installation and maintenance.

In this paper, an extended version of a previous paper published by the authors [7] is presented, with a more detailed and novel description of a system based on WSN for retrieving real time environmental data in order to optimize irrigation timings. A larger test case is described, with more test scenarios, followed by new experimental results and a comparison with previous ones. Finally, the main conclusions and future work are also presented.

## 2. Related Work

As presented in the previous research, many projects [8,9,10,11] have been published. These implementations for IoT solutions that control irrigation systems, mainly using single nodes with Arduinos, combined with a set of environmental data sensors, have several problems due to the large amounts of wire used to spread them across the fields. All these projects use the collected data to increase the garden or agricultural field efficiency, including user comfort and water savings, but lack the ability to be implemented in a real case scenario.

More advanced projects were also found [12,13,14,15,16] where full WSNs are used to automatically turn on the irrigation system when low levels of soil moisture are detected. Although some good results can be found, none works with the goal of water management.

In terms of real water management, and not only turning the irrigation off when the soil is still moist, not many works were found. The SWAMP project, by C. Kamienski [17,18,19], was the main research found. The project consists of a full system, from software to hardware, that includes the use of sensors, drones and weather stations to retrieve data, LoRa and MQTT (Message Queuing Telemetry Transport) to transmit data and a set of models to adapt these values to create the best irrigation process for precision agriculture.

Our proposal, already tested on a small scale in a previous research [7], stands out from the others by the use of algorithms capable of analyzing the environmental data and understanding the correct amount of water needed for the field, instead of watering only when moisture is low. This allowed for savings of around 25%. Even so, the previously presented systems lack some reliability, mainly in the chosen sensors, and needed to be tested in a larger scenario. In order to improve the results and guarantee that the solution works in multiple environments, we present an improved proposal, with a more reliable WSN.

## 3. Materials and Methods

In order to achieve the goal of collecting garden information in real time, the proposed solution, bearing in mind the WSN fashion, is composed of a set of hardware nodes, responsible for the collection of sensor data, which communicate among each other using LoRa [20] and with the server via MQTT [21] over WiFi. Then, a set of Software algorithms complements the network to store and analyze the collected data.

The system architecture is based on a WSN that was previously developed and tested in several scenarios by the authors [7,22,23,24]. The WSN is composed of a set of two different nodes, each with their responsibilities and features:

- The aggregation node: a single gateway, responsible for maintaining the connected network and communicate with the server. It is responsible for receiving the messages sent from the sensor nodes and sending them to the server.
- The sensor nodes: multiple nodes, responsible for collecting sensor information and then transmitting them the server to be analyzed.

**Figure 1:**
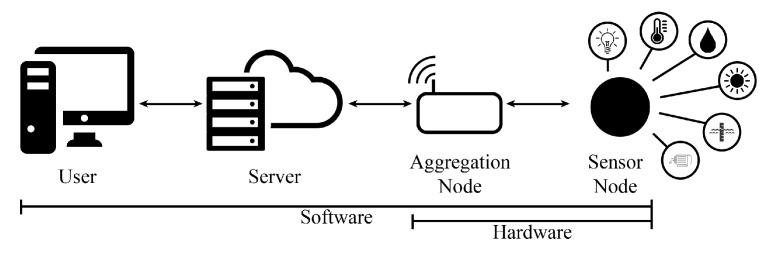
System Architecture [7] ©2019 IEEE.

A high-level view of the system architecture can be seen in Figure 1.

### 3.1. Hardware

As described by the authors in their previous research [7,22,23,24], each node is composed of an ESP32 ultra-low-power consumption microcontroller, with built-in WiFi and the ability to connect up to 18 analog sensors through its 12-bit Analogic Digital Converters (ADCs) [25], and an RFM95W, a transceiver LoRa radio module capable of creating encrypted multi-point network, that can communicate up to 2000 meters [26]. An 868MHz dipole flexible antenna is part of the hardware that forms the nodes.

Although the same hardware is used on both types of nodes, aggregation and sensors, each has specific characteristics. The built-in ESP32 WiFi connection is used by the aggregation node, however, in the sensor node, no WiFi connection is used, which allows this node to use the deep-sleep in order to run on batteries for a longer period of time, on a low-power fashion. The other difference from the nodes is that the sensor node is complemented with an array of sensors, composed of:

- Sensirion SHT30-DIS-P2.5KS: a high accurate and reliable sensor, capable of retrieving temperature and air humidity with an accuracy of ±0.1 °C and ±1.5%, respectively, via an Inter-Integrated Circuit ($I^{2}C$), was the perfect solution for our specifications, since it is low-cost, has a low power consumption with only 1.7 μA on average, and is easy to install and to retrieve data [27]. This sensor is a huge improvement when facing the DHT22 used before by the authors in [7], not only in terms of power consumption and size but also has a better accuracy;
- Capacitive moisture sensor: works by measuring the capacitance changes between the dielectric plates, and is capable of detecting the amount of water in the soil. The advantages of these types of sensors, not only include a better reading of soil moisture, but also avoid corrosion.

**Figure 2:**
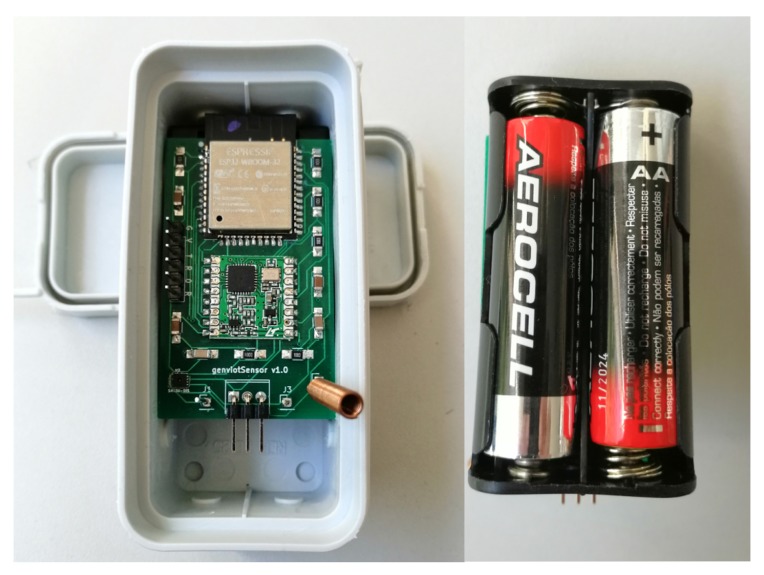
Smart Node.

Both types of nodes are enclosed in an IP68 box, as shown in Figure 2, to guarantee that environmental conditions such as rain, wind, heat or dust and wildlife, do not damage the electronics when placed in the implementation environment.

The system was developed to be power efficient and run on batteries for a long period of time, while being able to collect and transmit information. In this case, only the sensor node can be powered by batteries, since it is the only one that enters in deep sleep after collecting and transmitting the sensor data. The aggregation node, since it needs to always be listening to new sensor messages, is powered by a 5 V power adapter and a voltage regulator.

The sensor node power consumption in its life cycle can be found in Table 1.

Since the sensor node is in deep sleep mode for almost 90% of each collecting cycle, and due to the components chosen, the node can be powered by 2 AA batteries, as shown in Figure 2, for long periods of time. Table 2 presents the expected life-cycle of the batteries, based on real tests, depending on the interval between sample collection.

The proposed hardware architecture for the WSN meets the requirements of the current IoT systems and proves to be an optimization from the current solutions on the market, since it is able to adapt to multiple situations, as shown by the authors in their previous research and tests [7,22,23,24]. Besides that, the ability to perform in a low-power scheme and also adapt the consumptions to the user needs, allows the system to be very reliable in terms of battery replacements.

The proposed system can support a network of up to 250 nodes, with bigger networks not compromising the reliability of the system nor giving extra work in the implementation, since the nodes are configured automatically inside the network when booting for the first time.

### 3.2. Communication

Communication is the most important part of a WSN, being responsible not only for transmitting the collected data, but also to keep the network connected, create reliability and guarantee that the system can perform the tasks that were specified. In a WSN, communication also helps improve the coverage of the implementation environment, mainly with new protocols, such as LoRa or SigFox, allowing the network to expand up to 5 km.

Although only two types of nodes exist in the network, two communication protocols are required to allow the network to work, since an intra-network communication for communication between the network nodes, and an Internet communication to send data to the servers, are needed. Both networks will be described in the next sections.

#### 3.2.1. Node-to-Node Communication

This is how information is exchanged inside the WSN, from sensor information to actuator actions. Since nodes can be far from each other, and as some of the nodes are powered by batteries, a wireless communication capable of long-range transmissions with low power consumption is needed.

LoRa, due to its long range and low power consumption and also the fact that it works on an unlicensed radio spectrum [20], meaning that no costs are associated, was the chosen communication standard to connect the WSN. With the ability to eliminate repeaters, reduce device cost, increase battery lifetime on devices, improve network capacity and support a large number of devices, when facing other major communication protocols, as is presented in Table 3 [20,28,29,30,31,32,33,34,35,36,37,38,39,40,41], make LoRa the ideal solution to connect our WSN.

Hence, in order to connect all the network nodes, a LoRa peer-to-peer network was implemented, using the RFM95W radio transceiver. These modules, capable of using the LoRa protocol, allow the network to cover up to 2 km with up to 256 nodes, with individual node addressing [26]. With the LoRa radio module only consuming 70 mA while transmitting and with a sleep mode feature, they check all the necessary specifications in terms of node-to-node communication.

For configuration and usage, the Arduino library RadioHead [42] was used; this enables the node addressing, with a unique address being chosen, guaranteeing that each node has its own address. The nodes can communicate in two different ways, both encrypted by the library:

- By sending a message directly to a specific node, using its destination address;
- Sending a broadcast message to every node in the network, including the destination node ID inside the message.

Network fidelity is assured by the RadioHead library, with the use of automatic acknowledgement (ACK) response to the node that sends the message using its ID, as in the first scenario. When a broadcasting message is sent, as for the second scenario, no ID is used and so, no ACK mechanism is used by the library. To cope with this limitation, an ID is added to the broadcasting message in order for the receiving node to send an ACK response to the transmitting node.

The first scenario is usually used for the sensor nodes to send data to the gateway, while when the gateway send information for the sensors or actuators, the second scenario is used.

#### 3.2.2. Node-to-Server Communication

This is how information containing sensor data leaves the network and actions are received. For that, some sort of Internet connection is needed to connect to the server, and since our core microcontroller, the ESP32, has built-in WiFi, we use that functionality to transfer data.

**Figure 3:**
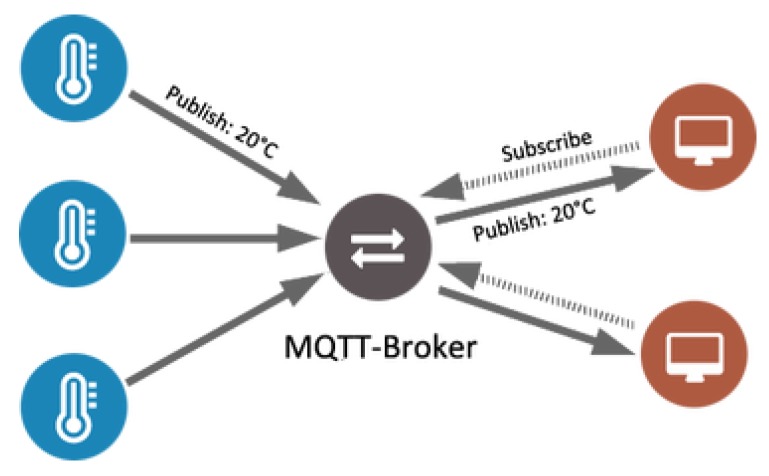
MQTT architecture.

In order to create a constant low-power data connection, the MQTT protocol was adopted, due to its high communication reach and the low power consumption, when compared to other protocols. MQTT is a messaging protocol [43,44,45] built on top of the TCP protocol, that uses a publish/subscribe pattern, as shown in Figure 3, to provide flexibility and simplicity transition making MQTT an optimal connection protocol for the IoT and M2M, being suitable for small, cheap, low power and low memory devices with low bandwidth networks.

To implement this protocol, it was necessary to define specific topics for each way of communication, making it possible to distinguish between messages intended for the server and those intended for the aggregation node:

- “irrigation/network/in” – to send messages to the server;
- “irrigation/network/out” – messages whose destination is the network.

The network, and particularly the aggregation node, subscribes to the “irrigation/network/out” topic and is constantly listening to new messages, for example, action to turn on/off the irrigation. When a message is received through the LoRa network from another node, for example, new sensor information, the aggregation node publishes that message to the topic “irrigation/network/in”. The server does the exact opposite, subscribing to the “irrigation/network/in” to wait for new sensor information, and publishing to “irrigation/network/out” when a new action is created.

#### 3.2.3. Security

As this will be implemented in real case scenarios, all the system communications must be secure, including the radio transmissions and the MQTT messages. For that, all messages are encrypted using a private key.

## 4. Implementation Scenario

**Figure 4:**
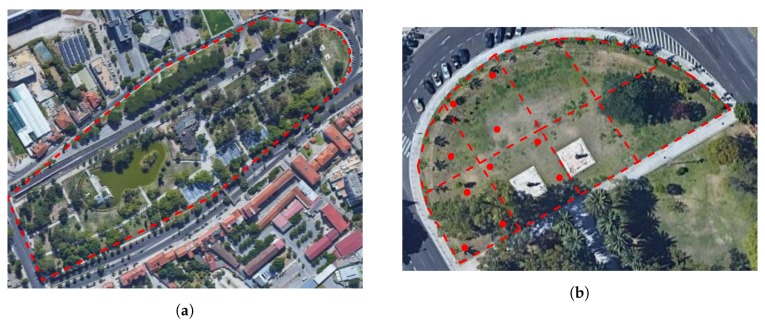
(**a**) Satellite Image of the Garden; (**b**) Irrigation zones and sensor implementation.

To test if the solution can work to improve convectional irrigation system efficiency, the implementation scenarios must contain a garden with an existing system. In the work developed previously by the authors in [7] a small garden with only three irrigation zones was chosen, so now the goal was to select a bigger garden, with more diversification of irrigation zones and also where people usually pass, in order to see if the system affects in any way the normal behaviour of the garden. The selected garden, as seen in Figure 4a, is a 2 ha public garden with more than 60 irrigation zones, in Campo Grande, Lisbon.

Since it was difficult to cover the entire garden with sensors, the northern side of the garden was chosen. As displayed in Figure 4b, this part of the garden has eight irrigation zones, each with approximately 350 $m^{2}$, and on four of them, we implement up to three sensor nodes, represented as red dots, depending on size and type of plants. One aggregation node was included in a nearby machine house, where a WiFi connection was available.

**Figure 5:**
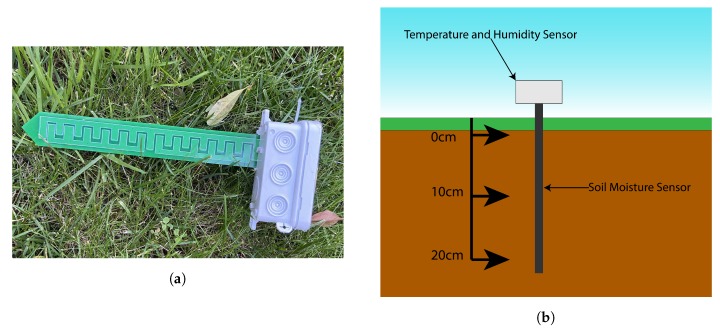
(**a**) Smart Node with Soil sensor; (**b**) Irrigation zones and sensor implementation.

Figure 5a,b shows how the sensor nodes were implemented in the irrigation zones, and mainly how the soil moisture sensor is capable of analysing multiple depths.

As in the previous research, our system does not replace a convectional irrigation system, in order to comprehend and compare the results with the use of real time environmental data.

Therefore, each sensor node ran for two months, in June and July, retrieving air temperature, relative humidity and soil moisture every 4 h and sending them to the servers via the aggregation node.

## 5. Results

**Figure 6:**
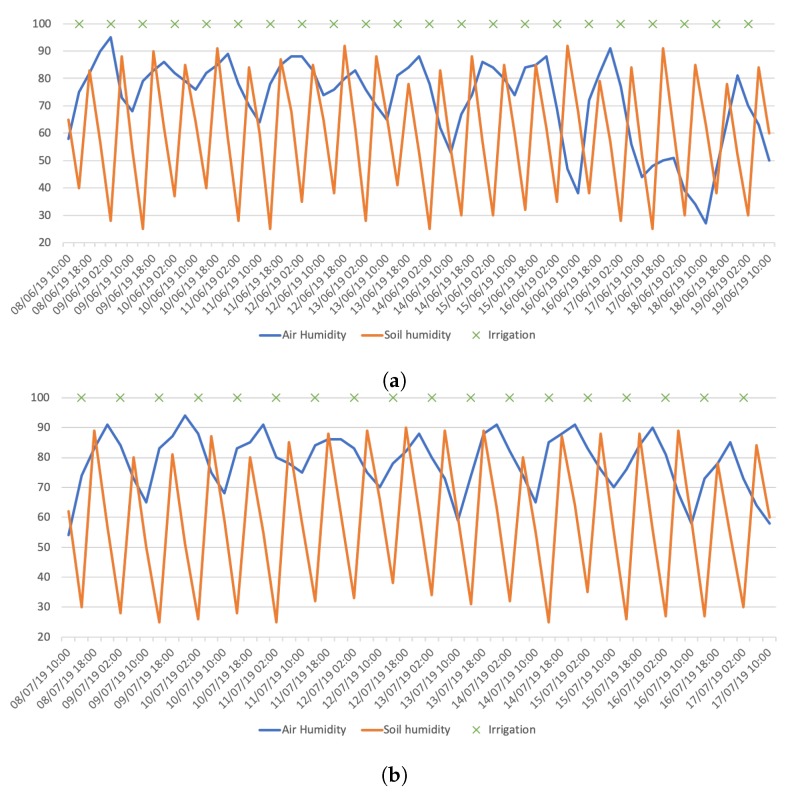
Data collected: (**a**) 2 week June data; (**b**) 2 week July data.

The system implementation allowed the collection of about 320 valid samples per sensor node, containing real time local information about the garden environmental parameters. Two sets of collected data can be seen in Figure 6, with 2 weeks in June represented in Figure 6a and 2 weeks in July represented in Figure 6b.

The first thing to notice is that in the testing period, there were no rain periods, and that irrigation was mainly done when soil moisture was below 30%, but it is also possible to notice that humidity levels are high, which helps the soil moisture to decrease more slowly, so the water used in the irrigation can be less.

It was possible to understand that the sensors spread throughout the garden do not have any implications in the normal activities in the garden, not harming the users, plants or do not even need to be removed when maintenance occurs in the garden, such as mowing the grass.

Analysing the obtained real time sensor data, it is possible to comprehend that these data can help improve the efficiency of the irrigation system.

The real water needs depend on the type of plants, the evapotranspiration, garden area, type of valves and tubing, distance between valves and the number of irrigations per day. Considering all these parameters, using Equation (1), it is possible to calculate the real amount of water for that particular garden.

(1) $$T=\frac{A\times (K_{c}+ET)\times 60}{F\times N\times 1000}/P$$

where *T* is the irrigation time in minutes, $K_{c}$ is crop coefficient, in this case for grass is 6 L/$m^{2}$, *F* is the outgoing flow of water per valve in $m^{3}$/h, *N* the number of valves, *P* is the number of irrigation periods, *A* is the garden area in $m^{2}$, given by Equation (2)

(2) $$A=\left[\left(0.5\times N\right)-1\right]\times D^{2}$$

where *D* is the distance between the valves, and ET is the evapotranspiration in mm/day, given by simplified Hargreaves formula [46] shown in Equation (3)

(3) $$ET=0.0023\times \left(T_{med}+17.78\right)R_{0}\times {\left(T_{max}-T_{min}\right)}^{0.5}$$

where $T_{med}$ is the average temperature, $T_{max}$ is the maximum temperature, $T_{min}$ is the minimum temperature and $R_{0}$ is the incident extra-terrestrial solar radiation which, based on latitude and time of the year, for Lisbon is about 17.1 mm/day.

It is possible to see that the only parameter in which variations occur is the value of temperature, both minimum and maximum, for a particular day. This is the first evidence that collecting local data is important.

To use as much of the collected environmental data as we can and to create a more efficient system, the irrigation times formula (Equation (1)) was adapted to take into consideration the last air humidity and soil moisture values. This new formula, Equation (4), allows to have a more precise irrigation time, since it includes all the environmental data that affect garden health.

(4) $$T_{opt}=TI_{soil}\times 0.7+TI_{hum}\times 0.3+T\times 0.1$$

where $TI_{x}$ is the time needed to reach 100% of the variable *x*, in this case soil moisture (soil) and air humidity (hum), given by Equation (5)

(5) $$TI_{x}=\frac{T\times (100-I_{x})}{100}$$

where $I_{x}$ is the last value retrieved for the sensor *x*.

The weight of each variable in the optimized formula can be adjusted from time to time, in order to reach a more precise formula. This, in the future, will be done using Machine Learning prediction algorithms. The 0.1 added in the end, is a margin of error, hence, the 1.1 total weight.

Considering the formulas obtained and how much data we can input to get better results, three analyses were done and compared to the normal irrigation system, already implemented in the garden:

1. Irrigation system controlled by our solution while using Equation (1) and forecast data to calculate the irrigation times;
2. Irrigation system controlled by our solution while using Equation (1) and sensor data to calculate the irrigation times;
3. Irrigation system controlled by our solution while using the optimized Equation (1) and sensor data to calculate the irrigation times;

With these scenarios, not only are we able to check if the use of sensor values can really help adjust the irrigation times but also prove that locally retrieved data are better than weather forecast for that region.

In the first scenario, the national Portuguese weather institute, IPMA [47], weather information was used, through their API, with the hourly temperature and the higher and lowest values for relative humidity for each day in the garden location. The values were retrieved from the IPMA API everyday at midnight, in order to get the values for the next 24 h.

**Figure 7:**
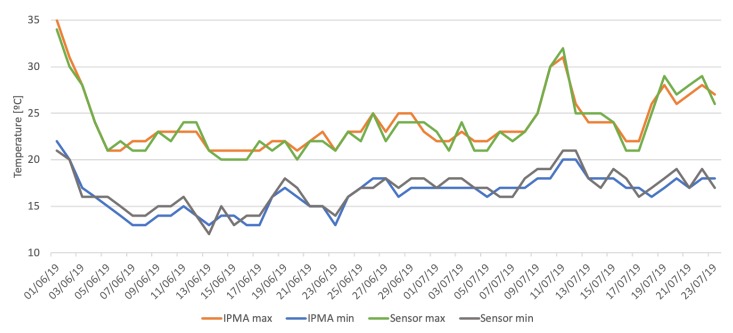
IPMA vs Sensor data comparison.

Figure 7 compares the IPMA weather forecast data with the collected data from the sensor network, and is possible to understand that although they follow the same pattern, some variations occur, that might affect the end result.

In the remaining scenarios, only the sensor network information was used. When an irrigation schedule approaches, the last needed values retrieved from the 4 hour interval datapoints are used in order to calculate the needs based on the most current conditions available.

Table 4 shows the irrigation times for each of the tested scenarios.

The obtained results shows that a viable solution that can reduce the amount of water used due to the adoption of a sensor data approach, is possible. To verify that by applying a solution based on the sensor data retrieved from the environment, the amount of time the valves are open are reduced, meaning that the amount of water used will be lower while maintaining the garden healthy.

Since valves are open for a reduced amount of time, less water will be used, while keeping the garden healthy. Equation (6) can be used to calculate the amount of water used based on the time that the valves need to be open.

(6) $$C=\frac{(F\times N\times 1000)\times T}{60}$$

This allows a comparison between the consumptions of the normal irrigation system and the theoretical consumptions of a system based on our solution. Table 5 shows the amount of water used in the scenarios described above.

With these results, it is possible to conclude two major results. Firstly, using data to set the irrigation times allows a reduction in up to 34% of the water used to irrigate a garden. This shows that a proper analysis of the environment can help create a more efficient and sustainable irrigation service, not only saving water but also reducing the costs for the owner.

Besides that, and bearing in mind the goal of this paper of assessing if real time and local data can offer improvements over historical or forecast data, it is possible to see that when using the collected data over the IPMA weather data, an improvement of almost 2% is possible. This might seem like a low improvement, but when we put it into perspective, it relates to almost 10,000 L/h saved.

Finally, it is possible to conclude that when more parameters are used, the best results are obtained. Using the optimized Equation (1), that uses soil moisture and air humidity besides only air temperature, we get an 8% improvement facing the normal Equation (1).

Maintaining the irrigation hours, but using the real amount of water that the garden needs, it is possible to get the same results in terms of soil moisture and garden health, while reducing water consumption.

## 6. Conclusions

In this article, the improvements done on a previous published research [7] are presented, with the main goal of creating a reliable WSN system to collect real time environmental data to optimize irrigation times. This paper shows the hardware and software improvements as well as the results from a new real case implementation in a bigger garden and for a longer period of time.

In the previous research, it was possible to conclude that the retrieved data were could be introduced in an intelligent and efficient algorithm in order to predict a more accurate irrigation timing. It was also possible to understand that by keeping track of environmental status, such as rain or high humidity values, it is possible to detect if it is really necessary to irrigate. In that case study, an improvement of 25% was achieved, although implementation was done in a small garden and for only 2 weeks.

The results from this new case study show again that using local real time data is a big advantage when facing forecast and historical data, with improvements of 2%. When using data facing a typical irrigation system, the improvements go up to 26%, when using just temperature, and 34% when combining more sensor data, such as humidity and soil moisture.

Once again, this number can be improved to reach 50% with some modifications to the irrigation system, namely, the valves and tubing used as well as the application layout.

It is also important to note that the savings are theoretical, since the irrigation controller was not changed. It is possible to evaluate the real amount of water saved, since this can be affected by numerous situations.

By combining the sensor data gathered by the WSN with Artificial Intelligence algorithms, it possible to configure, in real time, the WSN to adapt to new conditions or specifications, but also achieve a better efficiency in the process leading to savings for the final user, not only monetary but also in natural resources, such as water and energy, leading to a more sustainable environment.

The next step of this project is to use Machine Learning in order to predict the future conditions of the garden and prepare for drought periods or other environmental conditions that can affect the garden. We also aim to extend the application to agriculture, where there is might be a large number of crops, each with different water needs that would affect irrigation times.

## Figures and Tables

**Table 1:** Sensor node power consumption.

Node Status	Power Consumption $V_{IN}$ = 3 V
Transmitting	80 mA
Collecting data	20 mA
Deep Sleep	100 μA

**Table 2:** Sensor node power consumption.

Interval	Battery Life-Cycle
10 min	2 months
30 min	5 months
1 h	13 months
4 h	±3 years
12 h	±5 years
24 h	±10 years

**Table 3:** Major Communication Wireless Protocols Characteristics.

Feature	Wi-Fi	Bluetooth	ZigBee	LoRa	SigFox	NB-IoT
Data Rate (kbps)	11 × 10^3^	1 × 10^3^	250	110	1 × 10^−3^	250
Frequency (GHz)	2.4	2.4	2.4	0.868	0.868	1.8
Range (m)	1–100	10–100	10–100	5000	10,000	1000
Nodes/Master	32	7	65,540	15,000	-	-
Power Consumption $V_{IN}$ = 3.3 V [mA]	100–350	1–35	1–10	1–10	1–10	1–100
Security	WPA/WPA2	128 bit	128 bit	128 bit	-	128 bit

**Table 4:** Average irrigation time per scenario.

Scenario	Average Irrigation Time (min)
Current system	42
(1)	33.6
(2)	32.3
(3)	28.7

**Table 5:** Water used per scenario.

Scenario	Water Used (L/h)	Savings
(a)	614376	-
(b)	463561	24.55%
(c)	452017	26.43%
(c)	401541	34.64%
